# Myokines as Possible Therapeutic Targets in Cancer Cachexia

**DOI:** 10.1155/2018/8260742

**Published:** 2018-10-22

**Authors:** Emilia Manole, Laura C. Ceafalan, Bogdan O. Popescu, Carmen Dumitru, Alexandra E. Bastian

**Affiliations:** ^1^Molecular Biology Department, “Victor Babeș” National Institute of Pathology, Bucharest, Romania; ^2^Research Center, Pathology Department, Colentina Clinical Hospital, Bucharest, Romania; ^3^Ultrastructural Pathology Laboratory, “Victor Babeș” National Institute of Pathology, Bucharest, Romania; ^4^Department of Cellular & Molecular Biology and Histology, School of Medicine, “Carol Davila” University of Medicine and Pharmacy, Bucharest, Romania; ^5^Department of Neurology, School of Medicine, “Carol Davila” University of Medicine and Pharmacy, Bucharest, Romania; ^6^“Carol Davila” University of Medicine and Pharmacy, Bucharest, Romania; ^7^Pathology Department, Colentina Clinical Hospital, Bucharest, Romania

## Abstract

Cachexia is an extremely serious syndrome which occurs in most patients with different cancers, and it is characterized by systemic inflammation, a negative protein and energy balance, and involuntary loss of body mass. This syndrome has a dramatic impact on the patient's quality of life, and it is also associated with a low response to chemotherapy leading to a decrease in survival. Despite this, cachexia is still underestimated and often untreated. New research is needed in this area to understand this complex phenomenon and ultimately find treatment methods and therapeutic targets. The skeletal muscle can act as an endocrine organ. Signaling between muscles and other systems is done through myokines, cytokines, and proteins produced and released by myocytes. In this review, we would like to draw attention to some of the most important myokines that could have potential as biomarkers and therapeutic targets: myostatin, irisin, myonectin, decorin, fibroblast growth factor 21, interleukin-6, interleukin-8, and interleukin-15.

## 1. Introduction

Cachexia is an extremely serious syndrome manifested by anorexia, weight loss through loss of muscle mass and fatty tissue, inflammation, and increased energy consumption that occurs in many chronic diseases, of which cancer occupies a special place (80% of patients with cancers develop cachexia) [1]. Cachexia occurs in most patients with terminal cancer and is responsible for death of approximately 22% of patients [2]. It is characterized by systemic inflammation, a negative protein and energy balance, and involuntary loss of body mass. This syndrome has a dramatic impact on the patient's quality of life, and it is also associated with a low response to chemotherapy and leads to a decrease in survival [3–5].

Cachexia is still underestimated and often untreated [6, 7] despite its association with many mechanisms, especially inflammatory, which contribute to the installation of a persistent catabolic status.

The current strategy focuses on treating cancer, with the hope that it will completely reverse cachexia syndrome. But this is not valid in advanced cancers. Another option is to increase nutritional intake, but the anorexia of cachectic patients is only part of the problem, nutrition as unimodal therapy not yielding the expected results. In addition, radiochemistry may exacerbate the progression of cachexia in a number of patients [8, 9].

Until ten years ago, cachexia was seen as an untreatable syndrome. In recent years, however, the management of cancer cachexia has greatly improved, as studies on the involved mechanisms have developed. Current treatment of cachexia in malignant neoplasm is a palliative one. Many anticancer products may have beneficial effects in treating cancer but worsen cachexia [10]. New research is needed in this area to understand this complex phenomenon and ultimately find treatment methods, therapeutic targets that prevent cancer progression but also improve the quality of patient's life. A multidisciplinary approach to treating cachexia would be necessary: new pharmacological agents combined with diet modification and exercise.

There are papers showing that the skeletal muscle can act as an endocrine organ [11, 12], exerting its influence on other organs/systems, maintaining physical activity and ultimately life. It is a tissue energy producer and consumer that influences the energy metabolism of the whole organism. Signaling between muscles and other systems is done through myokines, bioactive substances released by the skeletal muscles [13]. These muscle cytokines exert an autocrine, paracrine, and endocrine effect and play a role as metabolic mediators between muscle tissue and other tissues such as the adipose tissue [14, 15], cardiac muscle [16], liver [17, 18], and pancreas [18].

In this review, we will refer to myokines, one of the components of this complex mechanism that leads to the appearance of muscle weakness and muscle mass loss in cancer, that have an important potential to become therapeutic targets.

## 2. What Are Myokines

Myokines have been defined as cytokines and proteins produced and released by myocytes [19] under the action of contractile activity [12]. They exert an autocrine, paracrine, or endocrine effect. Their receptors were found in the muscle, fat, liver, pancreas, bone tissue, heart, brain, and immune cells [20]. For the role of muscle tissue as “endocrine organ,” there are several studies that address this subject from different angles, not necessarily in cachexia. Thus, the existence of myokines as metabolic mediators between skeletal muscle and other organs during exercise to maintain a healthy status is shown by Schnyder and Handschin [12]. Other articles, describing the involvement of skeletal muscle in the development of aging-related pathologies, highlight the role of myokines in inducing or protecting these pathologies, depending on the secretion amount [21]. There are studies that show the role of myokines in the general metabolism of the body and how they interact with other organs [18]. Only few papers describe the role of myokines in cancer, precisely in cancer cachexia, which is an area recently approached. Dalamaga's editorial draws attention to the interaction between adipokines and myokines in the pathophysiology of cancer, making a review of literature data related to this subject [22, 23].

**Figure 1:**
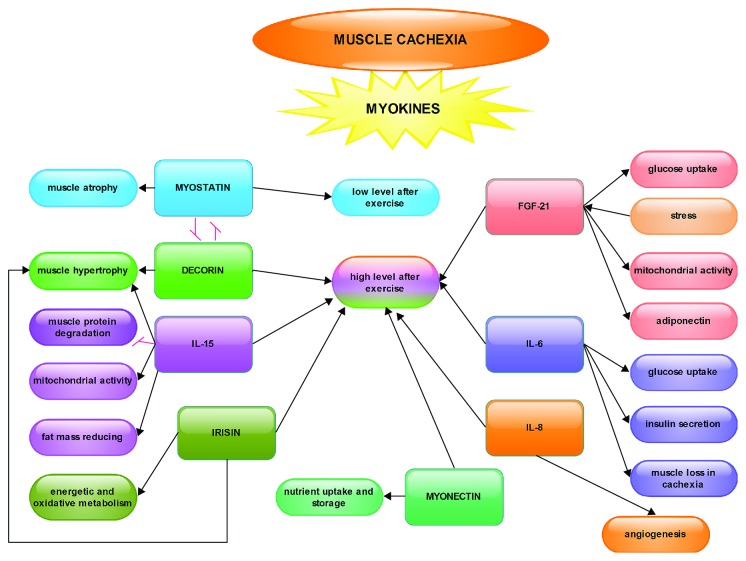
Effects of myokines in muscle cachexia. The schematic representation of myokine activity in the skeletal muscle shows the following: except for myostatin, which decreases after exercise, all others have a higher level after effort; between myostatin and decorin, there is an antagonistic relationship of mutual inhibition; the arrows show an activation or stimulation relationship between myokines and various metabolic processes that occur in the skeletal muscle.

For the reasons above, myokines are essential therapeutic targets in cachexia and the modulation of their expression could improve the maintenance of skeletal muscles at parameters as close as normal in cancer patients (Figure 1).

Without going into the details about the signaling pathways in myocytes, already described in other publications, we would like to draw attention to some of the most important myokines that would have potential as biomarkers and therapeutic targets.

### 2.1. Myokines as Potential Therapeutic Targets

The main myokines studied to date are myostatin, decorin, irisin, myonectin, interleukin-6 (IL-6), interleukin-8 (IL-8), interleukin-15 (IL-15), follistatin, fibroblast growth factor 21 (FGF21), bone morphogenetic protein (BMP), and brain-derived neurotrophic factor (BDNF) [12, 13, 22, 23]. Other possible factors have been detected in skeletal muscle, but their function, as well as their presence in the circulation, are largely unknown: musclin, nonneuronal acetylcholine [11].

Between these myokines, we would like to draw attention on some of the most studied so far (Table 1).

#### 2.1.1. Myostatin

Also called growth differentiation factor 8 (GDF-8), it is a member of the transforming growth factor-*β* (TGF-*β*) family, expressed in developing and adult muscular tissue. It is one of the first described myokines. Contrary to other myokines, which have a high level after exercise, myostatin has a low level after sustained muscular effort [24–29].

Its main function is the negative regulation of the muscle mass [30], which means high level of myostatin, less muscle mass. It plays a role in stopping myoblast proliferation and suppressing satellite cell activation, inducing muscle atrophy [31]. In addition, it influences the differentiation of muscle fibers by types (fast and slow) [32] and the arrangement of muscle glucose [33] as well as the muscle-adipose tissue cross-talking [34].

Myostatin influences the physiology of adipocytes, but it seems in an indirect manner. Pharmacological administration of myostatin *in vivo* and *in vitro* models does not lead to the reduction of adipose tissue by lipolysis [35].

It seems that in myostatin null mice, reduced body fat is caused especially by muscle mass growth. Myostatin null mice develop a massive muscular hypertrophy resulting from an accelerated myogenesis [21, 36], accompanied by a massive reduction in fatty tissue [30]. A similar phenotype has been described in a child with a mutation in the myostatin gene [37].

Interestingly for our subject, cachexia, is that the circulating leptin level, the “satiety hormone,” secreted by adipocytes, is reduced in mice with myostatin deficiency, although food intakes compared to control mice (WT) were not different [36, 38].

Although there are relatively few studies on the expression of myostatin in muscle cachexia, especially as a biomarker and therapeutic target, we consider it to be a good research approach in cachexia treatment, especially in conjunction with decorin and leptin.

#### 2.1.2. Irisin

Discovered in 2012 as a transmembrane protein [39], FNDC5 has a cleaved soluble form, irisin, that it is released into circulation during the proteolytic process after acutely exercising of skeletal muscles. It increases the energetic and oxidative metabolism of the muscle by activating genes related to these processes. It has a high level during myogenesis and induces glucose uptake [40], improving glucose homeostasis, inhibiting lipid accumulation, and reducing body weight [41].

It has been studied especially in relation to obesity but also with myopathies such as muscular dystrophy. In these latter studies, injection of irisin induced muscle hypertrophy, improving muscle strength and reducing necrosis and development of connective tissue in a murine model [42]. This study may be a starting point for attempts at therapeutic irisin targeting cancer cachexia as well.

#### 2.1.3. Myonectin (CTRP15)

Myonectin is a protein belonging to the C1q/TNF-related protein (CTRP) family, and it is found mainly in muscle, less in circulation, being especially related to nutritional metabolism. Thus, the expression of myonectin is stimulated by exercise and nutrients and is supposed to induce nutrient uptake and storage in other tissues, such as adipose tissue, causing a flux of glucose or fatty acids [43, 44].

It is less studied in connection with cachexia. We suppose that it could be a therapeutic target, just like other myokines, being linked to nutrient uptake.

#### 2.1.4. Decorin

Decorin is a small leucine-rich proteoglycan released by myotubes, and as other myokines, its circulating level is increased after acute exercise. Decorin is overexpressed in the skeletal muscle in humans and mice after chronic training [45]. It directly binds myostatin which is a strong inhibitor of muscle growth [36]. Decorin acts antagonistically to myostatin and is involved in restructuring muscle during hypertrophy [45].

Considering all of this, we can say that this myokine could be taking into account as the therapeutic target along with myostatin, being able to modulate the maintenance of muscle mass in cachexia.

#### 2.1.5. Fibroblast Growth Factor 21 (FGF 21)

Fibroblast growth factors are present in many tissues as signaling proteins and are implied in development and metabolism [46]. In the skeletal muscle, it has been shown that FGF21 has a role in glucose uptake in myotubes [47].

FGF21, as a myokine, is induced by stress [48]. Mitochondrial dysfunction after an autophagy deficiency increases the FGF21 level to protect against obesity induced by diet and insulin resistance [49]. In the mitochondrial respiratory chain deficiency, there is a compensatory increase in FGF21 level resulting in an increase in mitochondrial activity [50].

There is a close link between FGF21 and adiponectin that acts as downstream effector of FGF21, controlling in an endocrine mode the lipid homeostasis and glucose in the skeletal muscle and other organs, such as the liver. In turn, adiponectin regulates the influence of FGF21 on energetic metabolism and insulin sensitivity [51, 52].

FGF21 is a very poorly addressed myokine in the study of cachexia, although its involvement in the energy metabolism of the myocyte is demonstrated. Future research would be wanted to highlight its potential in therapeutic strategies as long as the energy metabolism of the muscle is very important in maintaining a normal state of this tissue.

#### 2.1.6. Interleukin-6 (IL-6)

IL-6 is the first myokine that has been discovered in the bloodstream, secreted by muscle cells after contraction [19], and one of the most studied.

It was originally described as a prototypic proinflammatory cytokine, then having anti-inflammatory properties also [53]. IL-6 is released by the immune system cells (monocytes/macrophages), fibroblasts, and endothelial cells [54] and also by the skeletal muscle correlated with the exercise [54–57]. Following the release of IL-6 by the muscle, it increased glucose uptake, oxidation of fatty acid, and insulin secretion. Although its release was originally linked to muscle damage [58], subsequently, a plasma increase in IL-6, less dramatic and nondamaging, was demonstrated in concentric muscular contraction and even immediately after exercise [19].

But how does IL-6 bind to cachexia and what therapeutic role can it have? A review on this subject was made by Narsale and Carson [59]. The authors show that IL-6 remains a promising therapeutic strategy for diminishing cachexia in many types of cancers. However, it is necessary to better understand the direct and indirect effects of IL-6, as well as its specific tissue actions to improve this treatment.

It is clear that diminishing this myokine can alleviate the progression of cachexia in cancer patients [60].

Numerous *in vivo* studies on rodents have been conducted to establish the mechanisms for muscle wasting producing. It has shown that there is a suppression of protein synthesis on the one hand and the activation of pathways of protein degradation on the other hand [61–64]. The muscle loss in cancer cachexia is directly or indirectly linked to overexpression of IL-6 [65–67]. But between the results obtained on murine cachexia models in different types of cancers, there are differences: in IL-6 mechanisms of action and in inhibition of various IL-6-dependent signaling pathways [68, 69] by attenuating or eradicating the progression of cachexia [67].

Unlike *in vivo* and *in vitro* investigations, studies on muscle mass recovery pathways in cancer patients are difficult to do, and the results differ from one type of cancer to another. It is certain, however, that advanced or terminal cancer patients have high levels of IL-6 in plasma, correlated with weight loss, anemia, and depression [70]. Clinical studies of an IL-6R inhibitor that inhibits the binding of IL-6 to its receptor, tocilizumab, have shown in patients with cancer cachexia the reduction of plasma IL-6 levels, the alleviation of muscle mass loss without affecting tumor proliferation [8, 71, 72]. Possible side-effects of suppression of interleukins, such as IL-6, which may be compromising patients' immune response to infections, should be monitored. Also, the effects of IL-6 signaling in organs other than muscles, such as liver and gut, should be considered [73].

#### 2.1.7. Interleukin-8 (IL-8)

IL-8 is a chemokine produced by muscle cells and also by other cells like macrophages, epithelial cells, and endothelial cells. It is a member of the CXC cytokine family and was originally described as a chemoattractant for lymphocytes and neutrophils [74, 75], and later, it was shown to be involved in angiogenesis and tumor growth [76].

In recent years, some researchers have shown that IL-8 is involved in cachexia, finding an elevated level in the serum of patients with this syndrome [77, 78], but rather like cytokine rather than myokine.

An additional argument that IL-8 plays a role in cachexia is brought by a publication that has shown that the genetic polymorphism of this myokine can contribute to the pathogenesis of cachexia in gastric cancer [79].

A team of researchers found IL-8 in the muscle, not the plasma, following exercise, indicating its local role in angiogenesis for example [80]. Although its physiological function is largely unknown, association with CXCR2 suggests its involvement in exercise-induced neovascularization in the muscle tissue [81].

It has been shown in healthy subjects that after muscle exercise, the level of myokines in the blood has increased. These include IL-8 and IL-15. Interestingly, a continuous muscle contraction with a moderate intensity induces a higher concentration of myokines than a shorter muscular contraction but with a high intensity [82]. This fact, correlated with the promotion of angiogenesis, could be a starting point for studies on IL-8 produced in muscular tissue as a therapeutic target in cancer cachexia and may be a key point in reducing muscle mass loss or in rebuilding skeletal muscle along with other factors.

Attention should also be paid to the fact that IL-8 is also produced in adipose tissue, especially the visceral one, and has a high level in obese patients [83]; the modulation of this myokine could be made from different directions/tissues.

#### 2.1.8. Interleukin-15 (IL-15)

IL-15 is present in the skeletal muscle, having an anabolic effect on the metabolism of muscle proteins, and is also modulated by exercise [20]. It decreases muscle protein degradation and reduces fat mass, playing an important role in skeletal muscle-adipose tissue interaction [84–88]. IL-15 overexpression induces muscle hypertrophy and is involved in the synthesis and inhibition of protein degradation as it is shown in an *in vitro* study [89].

This myokine is connected with the alteration of mitochondrial function, overexpression of muscle IL-15 increasing mitochondrial activity and adipose tissue mass [90].

The role of IL-15 in cachexia is not fully understood. An earlier study on a rat model with cancer cachexia showed that IL-15 decreases the rate of protein degradation without affecting protein synthesis [91]. A research conducted on adult patients with a diagnosis of recent cancer and weight loss showed that there was no difference between their serum IL-15 levels and those of healthy subjects [92].

Despite these controversial results, the potential of this interleukin is not excluded, and other studies are needed to show this.

An important idea that should be considered is that there are cytokines that can be released by both the immune system and the muscles. Inflammation occurs in cancer and may even induce cancer [93]. So when we act on the cytokines released by the muscles (myokines), we must also keep in mind that the same cytokines are released by other cells/tissues also, and they can be influenced by our action [94–96].

## 3. Conclusions

We cannot draw conclusions about the place and role of myokines in *cancer cachexia therapy* without reminding the complex pathophysiology they are involved in and the fact that there are many signaling pathways in this syndrome that interfere and interrelate.

One of these important *interactions is between skeletal muscle and adipose tissue*, more specifically between myokines, adipokines, and free fatty acids, as we have shown. It has been proven that 25% of cancers are caused by obesity and a sedentary life [97]. Myokines and adipokines play an essential role in maintaining the body muscle and body fat at normal levels and thus modulate the body composition [23, 98–100]. The lack of movement and the existence of a large adipose tissue contribute to the destruction of the skeletal muscle tissue that occurs in cancer cachexia.

*Cachexia treatment* may be a challenge because it is necessary to address multiple and complex determinant causes. It requires a therapeutic combination based on sustained research in the fields of pharmacology, nutritional intake stimulation, reduction of inflammation, etc. We would like to draw attention to the possibility of considering myokines as possible therapeutic targets. Some of them have already been considered, especially IL-6, but not all of them. They may be taken into account for targeted therapeutic interventions, especially in personalized medicine when specific tests could be performed for each patient regarding their specific released myokines, depending on the health status or muscle damage.

Consideration should be given to the possibility of cancer *cachexia prevention* also, so that patients could better respond to antitumor treatment. As we have seen, *exercise* can be a powerful tool in preventing and treating muscle cachexia, along with other therapies. Of course, the exercise should be moderate, not acute, in order to not interfere with unwanted metabolic changes in both skeletal muscle and other organs such as the liver, adipose tissue, and pancreas. Myokine expressions change after exercise and transmit signals from the muscles to the rest of the body. There are not many studies regarding this phenomenon in muscle cachexia, and research is needed in this direction to know how much exercise a cachectic patient needs to get beneficial effects in muscle recovery or even to prevent cancer cachexia.

## Figures and Tables

**Table 1:** The most studied myokines and their action mode in skeletal muscular tissue.

Myokine	Action	Level after muscle exercise
Myostatin	Stops myoblast proliferation Suppresses satellite cell activation Induces muscle atrophy	Lower level

Irisin	Activates genes related to oxidative metabolism Induces muscle hypertrophy Improves muscle strength Reduces necrosis	High level

Myonectin	Induces nutrient uptake Induces nutrient storage in adipose tissue	High level especially in muscle, less in circulation

Decorin	Acts antagonistically with myostatin Involved in restructuring muscle	High level

FGF21	Induces glucose uptake Increases mitochondrial activity Connected with adiponectin Implied in the control of lipid homeostasis, energetic metabolism, and insulin sensitivity	High level

IL-6	Increases glucose uptake, oxidation of fatty acids Increases insulin secretion Elevated in cancer cachexia—low level Alleviate cachexia progress	High level

IL-8	Elevated in cancer cachexia, especially like cytokine Induces angiogenesis	High level in muscle, not in plasma

IL-15	Anabolic effect Decreases muscle protein degradation Reduces fat mass Induces muscle hypertrophy Increases mitochondrial activity	High level

## References

[1] Fearon K., Strasser F., Anker S. D. (2011). Definition and classification of cancer cachexia: an international consensus. *The Lancet Oncology*.

[2] Warren S. (1932). The immediate causes of death in cancer. *The American Journal of the Medical Sciences*.

[3] Dewys W. D., Begg C., Lavin P. T. (1980). Prognostic effect of weight loss prior tochemotherapy in cancer patients. *The American Journal of Medicine*.

[4] Deans C., Wigmore S. J. (2005). Systemic inflammation, cachexia and prognosis in patients with cancer. *Current Opinion in Clinical Nutrition and Metabolic Care*.

[5] Tan B. H. L., Fearon K. C. H. (2008). Cachexia: prevalence and impact in medicine. *Current Opinion in Clinical Nutrition and Metabolic Care*.

[6] Evans W. J., Morley J. E., Argilés J. (2008). Cachexia: a new definition. *Clinical Nutrition*.

[7] von Haehling S., Anker S. D. (2010). Cachexia as a major underestimated and unmet medical need: facts and numbers. *Journal of Cachexia, Sarcopenia and Muscle*.

[8] Ando K., Takahashi F., Motojima S. (2013). Possible role for tocilizumab, an anti-interleukin-6 receptor antibody, in treating cancer cachexia. *Journal of Clinical Oncology*.

[9] Laine A., Iyengar P., Pandita T. K. (2013). The role of inflammatory pathways in cancer-associated cachexia and radiation resistance. *Molecular Cancer Research*.

[10] Aoyagi T., Terracina K. P., Raza A., Matsubara H., Takabe K. (2015). Cancer cachexia, mechanism and treatment. *World Journal of Gastrointestinal Oncology*.

[11] Iizuka K., Machida T., Hirafuji M. (2014). Skeletal muscle is an endocrine organ. *Journal of Pharmacological Sciences*.

[12] Schnyder S., Handschin C. (2015). Skeletal muscle as an endocrine organ: PGC-1*α*, myokines and exercise. *Bone*.

[13] Pedersen B. K. (2011). Muscles and their myokines. *Journal of Experimental Biology*.

[14] Trayhurn P., Drevon C. A., Eckel J. (2011). Secreted proteins from adipose tissue and skeletal muscle – adipokines, myokines and adipose/muscle cross-talk. *Archives of Physiology and Biochemistry*.

[15] Pedersen B. K., Febbraio M. A. (2012). Muscles, exercise and obesity: skeletal muscle as a secretory organ. *Nature Reviews Endocrinology*.

[16] Okita K., Kinugawa S., Tsutsui H. (2013). Exercise intolerance in chronic heart failure - skeletal muscle dysfunction and potential therapies. *Circulation Journal*.

[17] Gleeson M. (2000). Interleukins and exercise. *The Journal of Physiology*.

[18] Pedersen L., Hojman P. (2012). Muscle-to-organ cross talk mediated by myokines. *Adipocytes*.

[19] Pedersen B. K., Febbraio M. A. (2008). Muscle as an endocrine organ: focus on muscle-derived interleukin-6. *Physiological Reviews*.

[20] Pedersen B. K., Åkerström T. C. A., Nielsen A. R., Fischer C. P. (2007). Role of myokines in exercise and metabolism. *Journal of Applied Physiology*.

[21] Demontis F., Piccirillo R., Goldberg A. L., Perrimon N. (2013). The influence of skeletal muscle on systemic aging and lifespan. *Aging Cell*.

[22] Dalamaga M. (2013). Interplay of adipokines and myokines in cancer pathophysiology: emerging therapeutic implications. *World Journal of Experimental Medicine*.

[23] Li F., Li Y., Duan Y., Hu C. A. A., Tang Y., Yin Y. (2017). Myokines and adipokines: involvement in the crosstalk between skeletal muscle and adipose tissue. *Cytokine & Growth Factor Reviews*.

[24] Ruas J. L., White J. P., Rao R. R. (2012). A PGC-1*α* isoform induced by resistance training regulates skeletal muscle hypertrophy. *Cell*.

[25] Laurentino G. C., Ugrinowitsch C., Roschel H. (2012). Strength training with blood flow restriction diminishes myostatin gene expression. *Medicine & Science in Sports & Exercise*.

[26] Louis E., Raue U., Yang Y., Jemiolo B., Trappe S. (2007). Time course of proteolytic, cytokine, and myostatin gene expression after acute exercise in human skeletal muscle. *Journal of Applied Physiology*.

[27] Roth S. M., Martel G. F., Ferrell R. E., Metter E. J., Hurley B. F., Rogers M. A. (2003). Myostatin gene expression is reduced in humans with heavy-resistance strength training: a brief communication. *Experimental Biology and Medicine*.

[28] Mascher H., Tannerstedt J., Brink-Elfegoun T., Ekblom B., Gustafsson T., Blomstrand E. (2008). Repeated resistance exercise training induces different changes in mRNA expression of MAFbx and MuRF-1 in human skeletal muscle. *American Journal of Physiology-Endocrinology and Metabolism*.

[29] Saremi A., Gharakhanloo R., Sharghi S., Gharaati M. R., Larijani B., Omidfar K. (2010). Effects of oral creatine and resistance training on serum myostatin and GASP-1. *Molecular and Cellular Endocrinology*.

[30] McPherron A. C., Lawler A. M., Lee S.-J. (1997). Regulation of skeletal muscle mass in mice by a new TGF-p superfamily member. *Nature*.

[31] Joulia D., Bernardi H., Garandel V., Rabenoelina F., Vernus B., Cabello G. (2003). Mechanisms involved in the inhibition of myoblast proliferation and differentiation by myostatin. *Experimental Cell Research*.

[32] Wang M., Yu H., Kim Y. S., Bidwell C. A., Kuang S. (2012). Myostatin facilitates slow and inhibits fast myosin heavy chain expression during myogenic differentiation. *Biochemical and Biophysical Research Communications*.

[33] Cleasby M. E., Jarmin S., Eilers W. (2014). Local overexpression of the myostatin propeptide increases glucose transporter expression and enhances skeletal muscle glucose disposal. *American Journal of Physiology-Endocrinology and Metabolism*.

[34] Allen D. L., Cleary A. S., Speaker K. J. (2008). Myostatin, activin receptor IIb, and follistatin-like-3 gene expression are altered in adipose tissue and skeletal muscle of obese mice. *American Journal of Physiology-Endocrinology and Metabolism*.

[35] Stolz L. E., Li D., Qadri A., Jalenak M., Klaman L. D., Tobin J. F. (2008). Administration of myostatin does not alter fat mass in adult mice. *Diabetes, Obesity and Metabolism*.

[36] McPherron A. C., Lee S.-J. (2002). Suppression of body fat accumulation in myostatin-deficient mice. *The Journal of Clinical Investigation*.

[37] Schuelke M., Wagner K. R., Stolz L. E. (2004). Myostatin mutation associated with gross muscle hypertrophy in a child. *New England Journal of Medicine*.

[38] Lin J., Arnold H. B., Della-Fera M. A., Azain M. J., Hartzell D. L., Baile C. A. (2002). Myostatin knockout in mice increases myogenesis and decreases adipogenesis. *Biochemical and Biophysical Research Communications*.

[39] Boström P., Wu J., Jedrychowski M. P. (2012). A PGC1-*α*-dependent myokine that drives brown-fat-like development of white fat and thermogenesis. *Nature*.

[40] Lee H. J., Lee J. O., Kim N. (2015). Irisin, a novel myokine, regulates glucose uptake in skeletal muscle cells via AMPK. *Molecular Endocrinology*.

[41] Huh J. Y., Dincer F., Mesfum E., Mantzoros C. S. (2014). Irisin stimulates muscle growth-related genes and regulates adipocyte differentiation and metabolism in humans. *International Journal of Obesity*.

[42] Reza M. M., Sim C. M., Subramaniyam N. (2017). Irisin treatment improves healing of dystrophic skeletal muscle. *Oncotarget*.

[43] Seldin M. M., Peterson J. M., Byerly M. S., Wei Z., Wong G. W. (2012). Myonectin (CTRP15), a novel myokine that links skeletal muscle to systemic lipid homeostasis. *Journal of Biological Chemistry*.

[44] Seldin M. M., Wong G. W. (2012). Regulation of tissue crosstalk by skeletal muscle derived myonectin and other myokines. *Adipocytes*.

[45] Kanzleiter T., Rath M., Görgens S. W. (2014). The myokine decorin is regulated by contraction and involved in muscle hypertrophy. *Biochemical and Biophysical Research Communications*.

[46] Itoh N., Ornitz D. M. (2011). Fibroblast growth factors: from molecular evolution to roles in development, metabolism and disease. *Journal of Biochemistry*.

[47] Mashili F. L., Austin R. L., Deshmukh A. S. (2011). Direct effects of FGF21 on glucose uptake in human skeletal muscle: implications for type 2 diabetes and obesity. *Diabetes/Metabolism Research and Reviews*.

[48] Luo Y., McKeehan W. L. (2013). Stressed liver and muscle call on adipocytes with FGF21. *Frontiers in Endocrinology*.

[49] Kim K. H., Jeong Y. T., Oh H. (2013). Autophagy deficiency leads to protection from obesity and insulin resistance by inducing Fgf21 as a mitokine. *Nature Medicine*.

[50] Ji K., Zheng J., Lv J. (2015). Skeletal muscle increases FGF21 expression in mitochondrial disorders to compensate for energy metabolic insufficiency by activating the mTOR–YY1–PGC1*α* pathway. *Free Radical Biology & Medicine*.

[51] Lin Z., Tian H., Lam K. S. L. (2013). Adiponectin mediates the metabolic effects of FGF21 on glucose homeostasis and insulin sensitivity in mice. *Cell Metabolism*.

[52] Holland W. L., Adams A. C., Brozinick J. T. (2013). An FGF21-adiponectin-ceramide axis controls energy expenditure and insulin action in mice. *Cell Metabolism*.

[53] Kristiansen O. P., Mandrup-Poulsen T. (2005). Interleukin-6 and diabetes: the good, the bad, or the indifferent?. *Diabetes*.

[54] Akira S., Taga T., Kishimoto T. (1993). Interleukin-6 in biology and medicine. *Advances in Immunology*.

[55] Ostrowski K., Rohde T., Zacho M., Asp S., Pedersen B. K. (1998). Evidence that interleukin-6 is produced in human skeletal muscle during prolonged running. *The Journal of Physiology*.

[56] Jonsdottir I. H., Schjerling P., Ostrowski K., Asp S., Richter E. A., Pedersen B. K. (2000). Muscle contractions induce interleukin-6 mRNA production in rat skeletal muscles. *The Journal of Physiology*.

[57] Steensberg A., van Hall G., Osada T., Sacchetti M., Saltin B., Pedersen B. K. (2000). Production of interleukin-6 in contracting human skeletal muscles can account for the exercise- induced increase in plasma interleukin-6. *The Journal of Physiology*.

[58] Bruunsgaard H., Galbo H., Halkjaer-Kristensen J., Johansen T. L., MacLean D. A., Pedersen B. K. (1997). Exercise-induced increase in serum interleukin-6 in humans is related to muscle damage. *The Journal of Physiology*.

[59] Narsale A. A., Carson J. A. (2014). Role of interleukin-6 in cachexia: therapeutic implications. *Current Opinion in Supportive and Palliative Care*.

[60] Suh S. Y., Choi Y. S., Yeom C. H. (2013). Interleukin-6 but not tumour necrosis factor-alpha predicts survival in patients with advanced cancer. *Supportive Care in Cancer*.

[61] Puppa M. J., Murphy E. A., Fayad R., Hand G. A., Carson J. A. (2014). Cachectic skeletal muscle response to a novel bout of low-frequency stimulation. *Journal of Applied Physiology*.

[62] Penna F., Bonelli G., Baccino F. M., Costelli P. (2013). Mechanism-based therapeutic approaches to cachexia. *Vitamins & Hormones*.

[63] Suzuki H., Asakawa A., Amitani H., Nakamura N., Inui A. (2013). Cancer cachexia—pathophysiology and management. *Journal of Gastroenterology*.

[64] Gordon B. S., Kelleher A. R., Kimball S. R. (2013). Regulation of muscle protein synthesis and the effects of catabolic states. *The International Journal of Biochemistry & Cell Biology*.

[65] White J. P., Puppa M. J., Gao S., Sato S., Welle S. L., Carson J. A. (2013). Muscle mTORC1 suppression by IL-6 during cancer cachexia: a role for AMPK. *American Journal of Physiology-Endocrinology and Metabolism*.

[66] Bonetto A., Aydogdu T., Kunzevitzky N. (2011). STAT3 activation in skeletal muscle links muscle wasting and the acute phase response in cancer cachexia. *PLoS One*.

[67] Bonetto A., Aydogdu T., Jin X. (2012). JAK/STAT3 pathway inhibition blocks skeletal muscle wasting downstream of IL-6 and in experimental cancer cachexia. *American Journal of Physiology-Endocrinology and Metabolism*.

[68] Fearon K. C. H., Glass D. J., Guttridge D. C. (2012). Cancer cachexia: mediators, signaling, and metabolic pathways. *Cell Metabolism*.

[69] Onesti J. K., Guttridge D. C. (2014). Inflammation based regulation of cancer cachexia. *BioMed Research International*.

[70] Guo Y., Xu F., Lu T., Duan Z., Zhang Z. (2012). Interleukin-6 signaling pathway in targeted therapy for cancer. *Cancer Treatment Reviews*.

[71] Hirata H., Tetsumoto S., Kijima T. (2013). Favorable responses to tocilizumab in two patients with cancer-related cachexia. *Journal of Pain and Symptom Management*.

[72] Ando K., Takahashi F., Kato M. (2014). Tocilizumab, a proposed therapy for the cachexia of interleukin 6-expressing lung cancer. *PLoS One*.

[73] Berti A., Boccalatte F., Sabbadini M. G., Dagna L. (2013). Assessment of tocilizumab in the treatment of cancer cachexia. *Journal of Clinical Oncology*.

[74] Matsushima K., Morishita K., Yoshimura T. (1988). Molecular cloning of a human monocyte-derived neutrophil chemotactic factor (MDNCF) and the induction of MDNCF mRNA by interleukin 1 and tumor necrosis factor. *The Journal of Experimental Medicine*.

[75] Matsushima K., Baldwin E. T., Mukaida N. (1992). Interleukin-8 and MCAF: novel leukocyte recruitment and activating cytokines. *Chemical Immunology*.

[76] Maeda S., Ogura K., Yoshida H. (1998). Major virulence factors, VacA and CagA, are commonly positive in *Helicobacter pylori* isolates in Japan. *Gut*.

[77] Pfitzenmaier J., Vessella R., Higano C. S., Noteboom J. L., Wallace D., Corey E. (2003). Elevation of cytokine levels in cachectic patients with prostate carcinoma. *Cancer*.

[78] Krzystek-Korpacka M., Matusiewicz M., Diakowska D. (2007). Impact of weight loss on circulating IL-1, IL-6, IL-8, TNF-*α*, VEGF-A, VEGF–C and midkine in gastroesophageal cancer patients. *Clinical Biochemistry*.

[79] Bo S., Dianliang Z., Hongmei Z., Xinxiang W., Yanbing Z., Xiaobo L. (2010). Association of Interleukin-8 gene polymorphism with cachexia from patients with gastric cancer. *Journal of Interferon & Cytokine Research*.

[80] Szalay K., Rázga Z., Duda E. (1997). TNF inhibits myogenesis and downregulates the expression of myogenic regulatory factors myoD and myogenin. *European Journal of Cell Biology*.

[81] Frydelund-Larsen L., Penkowa M., Akerstrom T., Zankari A., Nielsen S., Pedersen B. K. (2007). Exercise induces interleukin-8 receptor (CXCR2) expression in human skeletal muscle. *Experimental Physiology*.

[82] Yeo N. H., Woo J., Shin K. O., Park J. Y., Kang S. (2012). The effects of different exercise intensity on myokine and angiogenesis factors. *The Journal of Sports Medicine and Physical Fitness*.

[83] Bruun J. M., Lihn A. S., Madan A. K. (2004). Higher production of IL-8 in visceral vs. subcutaneous adipose tissue. Implication of nonadipose cells in adipose tissue. *American Journal of Physiology-Endocrinology and Metabolism*.

[84] Li Y. H., Li F. N., Lin B. B., Kong X. F., Tang Y. L., Yin Y. L. (2014). Myokine IL-15 regulates the crosstalk of co-cultured porcine skeletal muscle satellite cells and preadipocytes. *Molecular Biology Reports*.

[85] Carbó N., López-Soriano J́., Costelli P. (2001). Interleukin-15 mediates reciprocal regulation of adipose and muscle mass: a potential role in body weight control. *Biochimica et Biophysica Acta (BBA) - General Subjects*.

[86] Nielsen A. R., Hojman P., Erikstrup C. (2008). Association between interleukin-15 and obesity: interleukin-15 as a potential regulator of fat mass. *The Journal of Clinical Endocrinology & Metabolism*.

[87] Quinn L. S., Anderson B. G., Strait-Bodey L., Stroud A. M., Argilés J. M. (2009). Oversecretion of interleukin-15 from skeletal muscle reduces adiposity. *American Journal of Physiology-Endocrinology and Metabolism*.

[88] Barra N. G., Reid S., MacKenzie R. (2010). Interleukin-15 contributes to the regulation of murine adipose tissue and human adipocytes. *Obesity*.

[89] Quinn L. S., Anderson B. G., Drivdahl R. H., Alvarez B., Argilés J. M. (2002). Overexpression of interleukin-15 induces skeletal muscle hypertrophy in vitro: implications for treatment of muscle wasting disorders. *Experimental Cell Research*.

[90] Barra N. G., Palanivel R., Denou E. (2014). Interleukin-15 modulates adipose tissue by altering mitochondrial mass and activity. *PLoS One*.

[91] Carbó N., López-Soriano J., Costelli P. (2000). Interleukin-15 antagonizes muscle protein waste in tumour-bearing rats. *British Journal of Cancer*.

[92] Martínez-Hernández P. L., Hernanz-Macías Á., Gómez-Candela C. (2012). Serum interleukin-15 levels in cancer patients with cachexia. *Oncology Reports*.

[93] Neagu M., Constantin C., Longo C. (2015). Chemokines in the melanoma metastasis biomarkers portrait. *Journal of Immunoassay and Immunochemistry*.

[94] Tanase C. P., Neagu M., Albulescu R. (2009). Key signaling molecules in pituitary tumors. *Expert Review of Molecular Diagnostics*.

[95] Pistol-Tanase C., Raducan E., Dima S. O. (2008). Assessment of soluble angiogenic markers in pancreatic cancer. *Biomarkers in Medicine*.

[96] Rutti S., Dusaulcy R., Hansen J. S. (2018). Angiogenin and osteoprotegerin are type II muscle specific myokines protecting pancreatic beta-cells against proinflammatory cytokines. *Scientific Reports*.

[97] McTiernan A. (2008). Mechanisms linking physical activity with cancer. *Nature Reviews Cancer*.

[98] Boone C., Mourot J., Grégoire F., Remacle C. (2000). The adipose conversion process: regulation by extracellular and intracellular factors. *Reproduction Nutrition Development*.

[99] Fruhbeck G., Gomez-Ambrosi J., Muruzabal F. J., Burrell M. A. (2001). The adipocyte: a model for integration of endocrine and metabolic signaling in energy metabolism regulation. *American Journal of Physiology-Endocrinology and Metabolism*.

[100] Diamond F. (2002). The endocrine function of adipose tissue. *Growth, Genetics & Hormones*.

